# Rupture of the Uterus

**Published:** 1900-01-20

**Authors:** 


### RUPTURE OF THE UTERUS.

At the last meeting of the Obstetrical Society Dr. Herbert Spencer read a paper on four cases of rupture of the uterus successfully treated by packing the tear *per vaginam* with iodoform gauze. They were the only cases in which he had used this method of treatment, and the only cases which he had known recover. All the others, about eight in number, had died in a few hours from shock and hæmorrhage, or in a few days from sepsis. Twice he had performed abdominal hysterectomy with a fatal result. He summarised his views in regard to the treatment of rupture of the uterus as follows: (1) Abdominal section is rarely required, and almost solely in cases where the fœtus has passed completely or in great part into the peritoneal cavity. When it is required it should be performed rapidly under local infiltration anæsthesia, and it should be followed by flushing of the peritoneal cavity with normal salt solution and suture of the tear, if possible, or, if this be not possible, by packing the tear with iodoform gauze and draining by the vagina or abdomen. (2) Abdominal hysterectomy is hardly ever necessary; when the broad ligaments are so much damaged as to endanger the vitality of the uterus vaginal hysterectomy should be performed. (3) All incomplete tears and most complete tears should be treated by packing the rupture *per vaginam* with iodoform gauze, after removing the clots and fluid blood. Several points were raised in the course of the discussion, one very important one being the statement by Dr. Horrocks that some of the success which he had seen follow the treatment of rupture of the uterus in recent years had been due, not entirely to the packing with gauze, but largely to the fact that the labours had been conducted aseptically. Another point was that raised by Dr. Herman to the effect that the suture of a ruptured uterus is useless unless the edges of the wound can be brought accurately together throughout its whole length. One or two sutures far apart were, he said, worse than useless, and in cases in which complete suture of the wound could not be done he thought packing with iodoform gauze was the best practice. The President (Mr. Doran), however, gave a warning about the use of iodoform gauze, saying that the absorption of iodoform caused rapid pulse, which might be taken as a symptom of sepsis. Many foreign authorities, he said, objected to any chemical antiseptics in packing wounds.

